# 

**Published:** 1886-04

**Authors:** 


					﻿AFRXL, 1886-
OLD SERIES
Vol. xxv
SERIES
b Voly411. -- S 0 2.'
1855
Mm®
1 EDIGAl^URME
JOURNAL!



’ W.F.WESTMORELAND.W®,
H.VM.M1LLER.M.D.LL .Djk
JAMES A.GRAY, M.D.
'PUBLISHED KY JAMES P.HARRISON&C®
jflP____________*&TLANTA,GA.' SiR
SUBSCRIPTION: $2.50 A YEAR.
				

